# Alcohol, metabolic risk and elevated serum gamma-glutamyl transferase (GGT) in Indigenous Australians

**DOI:** 10.1186/1471-2458-10-454

**Published:** 2010-08-03

**Authors:** Matthew T Haren, Ming Li, John Petkov, Robyn A McDermott

**Affiliations:** 1 Spencer Gulf Rural Health School (SGRHS), University of South Australia and The University of Adelaide, Whyalla Norrie SA, Australia; 2 Centre for Rural Health and Community Development (CRHaCD), University of South Australia, Whyalla Norrie SA, Australia; 3 Sansom Institute, Division of Health Sciences, University of South Australia, Adelaide SA, Australia; 4 Applied Statistics Unit, Centre for Regional Engagement, University of South Australia, Mount Gambier SA, Australia

## Abstract

**Background:**

The interaction between overweight/obesity and alcohol intake on liver enzyme concentrations have been demonstrated. No studies have yet examined the interaction between metabolic syndrome or multiple metabolic risk factors and alcohol intake on liver enzymes. The aim of this study was to examine if alcohol consumption modifies the effect of metabolic risk on elevated serum GGT in Indigenous Australians.

**Methods:**

Data were from N = 2609 Indigenous Australians who participated in a health screening program in rural far north Queensland in 1999-2000 (44.5% response rate). The individual and interactive effects of metabolic risk and alcohol drinking on elevated serum GGT concentrations (≥50 U/L) were analyzed using logistic regression.

**Results:**

Overall, 26% of the population had GGT≥50 U/L. Elevated GGT was associated with alcohol drinking (moderate drinking: OR 2.3 [95%CI 1.6 - 3.2]; risky drinking: OR 6.0 [4.4 - 8.2]), and with abdominal obesity (OR 3.7 [2.5 - 5.6]), adverse metabolic risk cluster profile (OR 3.4 [2.6 - 4.3]) and metabolic syndrome (OR 2.7 [2.1 - 3.5]) after adjustment for age, sex, ethnicity, smoking, physical activity and BMI. The associations of obesity and metabolic syndrome with elevated GGT were similar across alcohol drinking strata, but the association of an adverse metabolic risk cluster profile with elevated GGT was larger in risky drinkers (OR 4.9 [3.7 - 6.7]) than in moderate drinkers (OR 2.8 [1.6 - 4.9]) and abstainers (OR 1.6 [0.9 - 2.8]).

**Conclusions:**

In this Indigenous population, an adverse metabolic profile conferred three times the risk of elevated GGT in risky drinkers compared with abstainers, independent of sex and ethnicity. Community interventions need to target both determinants of the population's metabolic status and alcohol consumption to reduce the risk of elevated GGT.

## Background

Non-alcoholic fatty liver disease (NAFLD) has become the most common cause of chronic liver disease and cryptogenic cirrhosis in developed countries. The increasing prevalence of obesity, type 2 diabetes, and metabolic syndrome may be important drivers of increasing rates of NAFLD diagnoses [1]. An elevated serum gamma-glutamyl transferase (GGT) has been demonstrated as a biomarker of NAFLD [2] and, although it is not the only marker of chronic liver disease, it does predict future diabetes, coronary heart disease and stroke [3,4].

The Australian indigenous population has an excess of metabolic syndrome and type 2 diabetes relative to the non-indigenous population [5-7]. BMI-specific incidence rates for type 2 diabetes in Australian Aboriginal adults are among the highest in the world [8]. The overall incidence rate in rural Far North Queensland indigenous communities from 2000 to 2006 was 29 per 1000 person years (predicted to equate to 120 new diabetes diagnoses per year) [9]. Diabetes and CVD account for 7% and 14%, respectively of the overall burden of disease in Indigenous Australians, and for 12% and 24% respectively, of the health gap between indigenous and non-indigenous Australians [10].

Heavy and moderate drinkers are more likely to have elevated serum GGT when compared to abstainers [11]. The metabolic effects of alcohol include an increase in conversion of acetyl CoA to triglycerides (TG) which are secreted into the blood as very low density lipoproteins (VLDLs); ectopic fat accumulation in the liver from excess TG production and a rise in GGT and other liver enzymes; and weight gain from energy derived from alcohol (about 29 kj/gram) [12]. Alcohol accounted for 5% of the total Indigenous burden of disease, similar to high cholesterol and blood pressure, and for 4% in the health gap with non-indigenous Australians [10].

Li et al. reported elevated GGT with obesity, metabolic syndrome and physical inactivity in Indigenous non-drinkers in this cohort [13]. Little work has been published on NAFLD or alcohol-related liver disease in Indigenous populations worldwide. It is not known whether the additive effects of body fatness and alcohol intake on serum liver enzymes described in Nordic populations [14,15] apply to Indigenous Australians or whether drinking combined with multiple metabolic risk factors (or metabolic syndrome) further increases risk of elevated liver enzymes.

Evidence suggests that Aboriginal people have a greater risk of coronary heart disease events than would be expected based on Framingham risk functions [16]. Similar underestimation of risk using these algorithms has been reported in ethnic minorities in the UK [17] with overestimated risk in other ethnic groups [18,19]. Moreover, normative values for waist circumference have not been established for Aboriginal and Torres Strait Islanders (TSI) and these populations differ in a number of metabolic characteristics, relative to fatness [7,20,21]. Due to this uncertainty, we defined population metabolic profiles using cluster analysis and then compared the alcohol stratified effects of metabolic risk cluster versus International Diabetes Federation (IDF) classified metabolic syndrome on elevated GGT.

## Methods

### Population

Subjects were participants in The Well Person's Health Check [22]. Briefly, in 1999 - 2000, all indigenous residents aged 13 years and older, in 23 far north Queensland rural communities, were invited to participate through printed media, local radio, and word of mouth via the local health service, community council and community groups. A total of 3811 (participation rate 44.5%) participated in the study. The cohort was demographically representative of the indigenous population of the local area when compared to local population census data. Aboriginal and TSI are recognized as distinct ethnic groups and ascertainment of ethnicity was by self-identification in a manner consistent with Australian national minimum dataset specifications [23]. After exclusion of children under age 15 (N = 496), non-indigenous (N = 482) and individuals reporting joint Aboriginal-TSI descent (N = 224), a total of 2609 participants were included in this analysis. All participants provided informed consent to take part in the study. The study procedures were in accordance with the ethical standards on human research and thereby approved by the Cairns Base Hospital Human Research Ethics Committee with support from the peak Indigenous Health Organizations, Apunipima Cape York Health Council and the Torres Strait and Northern Peninsula Area Health Council.

### Data Collection

Height to the nearest 0.1 centimeter (cm) and weight to the nearest 0.1 kilogram (Kg) were measured in participants without shoes and wearing only light clothing. Body mass index (BMI) was calculated (weight (kg)/height (m^2^)). Waist circumference was measured midway between the lower border of the rib cage and the top of the iliac crest and recorded to the nearest 0.1 centimeter. Data were collected on self-reported medical conditions and none reported known liver disease. Physical activity was measured using a 7-day recall method in which the participants were asked to report daily physical activities of at least moderate intensity, lasting at least 30-minutes, performed during the week before their health check. Physical activity level was defined by American Heart Association criteria in which "active" reflects moderate to vigorous physical activity for at least 30-minutes per day on 5 days in the week before the survey [24]. Smoking behaviour was collected by standard questionnaire. The amount of alcohol consumed over a week was collected by 7-day recall diary and converted to number of standard drinks per week (1 standard drink = 10 grams of alcohol). Risky alcohol drinking was defined as greater than 6 standard drinks on any occasion or greater than 4 standard drinks per day in the recall week for males and greater than 4 drinks on any occasion or greater than 2 drinks per day in females [22,25].

Three seated blood pressure measurements were taken at 2 minute intervals after 10 minutes rest using a Dinamap automated oscillometric device (Critikon Corporation, USA) and the average was used in all analyses. Venous blood was sampled in the morning after an 8-hour fast as previously described [22]. GGT was measured using the kinetic photometric procedure with Cobas Integra 800 (Roche Diagnostics, USA). Blood glucose and lipids were measured using photometric enzyme endpoint assay with Cobas Integra 700/400 (Roche Diagnostics, USA).

The outcome variable, GGT, was classified as normal (<50 U/L) or elevated (≥50 U/L) based on laboratory specific reference ranges (Queensland Pathology Service, http://www.health.qld.gov.au/qhcss/qhps/default.asp). Metabolic syndrome was defined according to the IDF definition of waist circumference (Europid cut-offs: ≥94 cm for men or ≥80 cm for women) plus any two of the following: raised triglycerides (≥1.7 mmol/L); reduced HDL cholesterol (< 1.03 mmol/L for men or < 1.29 mmol/L for women); raised blood pressure (≥130 mmHg for systolic or ≥85 mmHg for diastolic); and raised fasting plasma glucose (≥5.6 mmol/L) [26]. Specific treatment for lipid abnormalities or previously diagnosed hypertension and previously diagnosed T2DM were not used in classification.

Waist circumference was used as a continuous variable and also to classify abdominal overweight and obesity, using World Health Organization (WHO) gender-specific criteria: overweight was ≥80 cm in women and ≥94 cm in men; obesity was ≥88 cm in women and ≥102 cm in men; < 80 cm in women and < 94 cm in men was classified as normal. BMI was used as a continuous variable and also to classify generalized overweight and obesity: < 25 Kg/m^2 ^(normal); 25-29.9 Kg/m^2 ^(overweight) and ≥30 Kg/m^2 ^(obesity) [27]. Diabetes was determined by clinical diagnosis of diabetes, verified by participants' medical records, or 2 hour glucose tolerance test or fasting blood glucose ≥7.0 mmol/L [28]. Blood pressure, fasting triglycerides, total cholesterol, HDL and glucose were used as continuous variables in all analyses.

#### Derivation of the metabolic risk factor cluster variable (MR cluster)

Cluster Analysis is the process of assigning members of a population into groups such that the members of each group share common characteristics. The method is an example of unsupervised learning. There is no *a priori *assumption made about what the groups represent or indeed the number of groups. Expectation Maximisation (EM) cluster analysis was applied to standardized (mean centred) variables: waist circumference, TG, HDL, systolic and diastolic blood pressure and fasting glucose, and was performed in STATISTICA (data analysis software system), version 8.0 (StatSoft, Inc. (2007) http://www.statsoft.com). To determine the major contributing variables to cluster membership, partial least squares (PLS) was applied since the predictors (the original variables) are highly correlated and so ordinary multinomial regression is inaccurate due to multicollinearity. Detail on both EM cluster analysis and PLS can be found in Additional file 1.

### Statistical Analysis

*A priori *it was expected that Aboriginal and TSI people would differ significantly in their anthropometric and metabolic characteristics, so comparisons for all variables were made across Aboriginal versus TSI ethnicity. Comparisons were also made for all variables across normal versus elevated GGT. Comparative analyses were by Chi-squared tests for categorical variables, independent t-tests for normally distributed continuous variables or Mann-Whitney rank-sum tests for non-normally distributed continuous variables. Unadjusted comparisons between MR clusters were analyzed using independent samples t-tests for continuous variables and Chi-squared analysis with Fisher's exact test for categorical variables. Logistic regression models were built to determine the associations (log odds) of drinking, metabolic syndrome and MR cluster with elevated GGT (≥50 U/L) for the whole population and stratified by ethnicity and sex. Similarly, odds ratios for the association of abdominal obesity, BMI categories, metabolic syndrome and MR cluster with elevated GGT, were calculated for the whole population and stratified by drinking status. Separate logistic regression models were built to examine the effect on elevated GGT of interactions between drinking and: sex; ethnicity; overweight and obesity defined by BMI and waist circumference; and metabolic risk defined by IDF MetS criteria and MR cluster. Models were adjusted for age, sex, ethnicity, BMI, smoking and physical activity levels where appropriate. Data analyses were performed using STATA version 10.1 (STATA Corp, College Station, Texas, USA).

## Results

Participants with missing data on serum GGT (N = 76) were not different in age, sex, BMI, metabolic profile or lifestyle behaviours than those with GGT data. Those missing information regarding metabolic syndrome (N = 224) were not different in age, sex, BMI, and lifestyle behaviours but had lower mean BP than those with complete metabolic syndrome information (125.5 ± 18.4 v 130.4 ± 19.6 mmHg, P < 0.001). Those missing records of drinking (N = 83) tended to be older (43.2 ± 17.6 v 37.2 ± 15.3 years, P < 0.001) and more likely to be female (65.1% v 51.4%, P = 0.014).

Table 1 describes the metabolic and behavioral characteristics of the cohort by Aboriginal and TSI status and sex. In both sexes, the TSI population had a significantly lower prevalence of elevated GGT, a higher prevalence of obesity, type 2 diabetes, metabolic syndrome and physically active people, and a lower proportion of smokers and drinkers, when compared with the Aboriginal population (all Ps < 0.05). Waist circumference, BMI, SBP and fasting glucose were higher and GGT and triglycerides were lower in TSI when compared with Aboriginal people (all Ps < 0.05). The prevalence of adverse MR cluster was significantly greater in TSI when compared to Aboriginal men, but was similar in women.

**Table 1:** Metabolic and behavioral characteristics of the study population, by ethnicity and sex

	Aboriginal (n = 1641)		TSI (n = 968)		Overall (N = 2609)
	**Mean or No**.	95% CI	**Mean or No**.	95% CI	**Mean or No**.
**Female**	N = 881		N = 471		
**Age**, years **(SD)**	37.4(15.5)	36.4- 38.4	38.3 (16.0)	36.9-39.75	37.7 (15.7)
**WC**, cm (SD) *	92.2 (16.5)	91.1- 93.3	103.6 (15.6)	102.1-105.1	96.2 (17.4)
**BMI**, kg/m^2 ^(SD)*	25.5 (6.6)	25.2-25.9	31.0 (6.7)	30.5- 31.4	27.6 (7.1)
**SBP**, mmHg (SD)*	125.5 (17.0)	124.1-126. 9	130.3 (16.1)	128.4-132.3	127.2 (21.3)
**DBP**, mmHg (SD)*	69.7 (13.5)	68.8-70.6	67.8 (12.2)	66.7-68.9	69.0 (13.1)
**T Chol**, mmol/L **(SD)**	4.8 (1.0)	4.7- 4.82	4.8 (1.0)	4.75- 4.9	4.8 (1.0)
**HDL**, mmol/L (SD) *	1.16 (0.3)	1.14-1.18	1.11 (0.2)	1.09-1.13	1.1 (0.3)
**GGT**, U/L (SD) *	39.2 (47.1)	36.0-42.4	27.2 (21.1)	25.3-29.1	35.0 (40.4)
**FPG**, mmol/L (SD)*	5.5 (2.5)	5.4-5.7	6.2 (3.1)	5.9-6.5	5.8 (2.7)
**Trigs**, mmol/L (SD)‡*	1.7 (1.2)	1.6-1.73	1.5 (1.1)	1.4- 1.6	1.6 (1.1)
Abdominal obesity † (%)*					
Normal	205 (23.6)	20.5-26.1	43 (9.1)	6.5-11.7	248 (18.5)
Overweight	158 (18.2)	15.4-20.4	40 (8.5)	6.0-11.0	198 (14.8)
Obesity	507 (58.3)	55.5-62.1	388 (82.4)	78.9-85.8	895 (66.7)
BMI (%)*					
<25	405 (46.0)	42.7-49.3	76 (16.1)	12.8-19.5	481 (35.6)
25-30	237 (26.9)	24.0-29.8	109 (23.1)	19.3-27.0	346 (25.6)
30+	239 (27.1)	24.2-30.1	286 (60.7)	48.8-55.1	525 (38.8)
Smokers (%)*	490 (56.0)	52.7-59.3	211 (44.8)	40.3-49.3	701 (52.1)
Drinking (%) *	526 (62.4)	59.2-65.7	233 (51.1)	46.5-55.7	759 (58.5)
Risky drinking † (%) *	356 (67.7)	63.7-71.7	112 (48.1)	41.6-54.5	468 (61.7)
Physically active † (%)*	164 (18.6)	16.0-21.2	117 (24.8)	20.9-28.7	281 (20.8)
GGT ≥50 U/L (%) *	181 (21.2)	18.4-23.9	36 (7.8)	5.4-10.3	217 (16.5)
Diabetes (%)*	114 (12.9 )	10.7-15.2	107 (22.7)	18.9-26.5	221 (16.4)
Metabolic syndrome † (%) *	353 (40.1)	36.9-43.4	246 (52.2)	47.7-56.7	595 (44.3)
**Adverse MR cluster profile † (%)**	268 (34.3)	31.0-37.6	171(37.7)	33.2-42.1	439 (35.6)

**Male**	N = 760		N = 497		
**Age**, years **(SD)**	36.6(14.9)	35.5-37.7	37.5 (15.2)	36.2-38. 9	37.0 (15.1)
**WC**, cm (SD) *	89.7 (14.6)	88.7-90.8	100.7 (15.0)	99.4-102.1	94.1 (15.7)
**BMI**, kg/m^2 ^(SD)*	24.5 (5.6)	24.1-24.9	29.8 (5.8)	29.2-30.3	26.6 (6.2)
**SBP**, mmHg (SD)*	130.8 (19.5)	129.6-132.0	136.3 (19.1)	134.8-137.7	130.0 (16.9)
**DBP**, mmHg **(SD)**	74.2 (13.8)	73.2-75.2	73.4 (13.1)	72.3-74.6	73.9 (13.5)
**T Chol**, mmol/L (SD)*	5.0 (1.1)	4.9- 5.04	5.2 (1.0)	5.1- 5.3	5.1 (1.1)
**HDL**, mmol/L (SD) *	1.2 (0.4)	1.17-1.22	1.1 (0.3)	1.102-1.15	1.2 (0.3)
**GGT**, U/L (SD) *	71.0 (85.6)	64.8-77.3	46.0 (49.2)	41.6-50.4	61.0 (74.2)
**FPG**, mmol/L (SD)*	5.4 (2.1)	5.2-5.5	6.0 (2.8)	5.7-6.2	5.6 (2.4)
**Trigs**, mmol/L (SD)‡*	2.1 (1.9)	2.0- 2.2	1.9 (1.4)	1.7-2.0	2.0 (1.8)
Abdominal obesity † (%)*					
Normal	486 (64.1)	60.5-67.4	156 (31.5)	27.3-35.5	642 (51.2)
Overweight	130 (17.2)	14.4-19.9	111 (22.4)	18.7-26.0	241 (19.2)
Obesity	142(18.7)	16.2-21.7	228 (46.1)	78.9-85.8	370 (29.5)
BMI (%)*					
<25	856 (52.2)	49.7-54.6	178 (18.4)	15.9-20.8	1034 (39.6)
25-30	417 (25.4)	23.3-27.5	287 (29.7)	26.8-32.5	704 (27.0)
30+	368 (22.4)	20.4-24.4	503 (52.0)	48.8-55.1	871 (33.4)
Smokers (%)*	516 (68.8)	65.5-72.1	274 (55.5)	51.1-59.9	1491 (57.6)
Drinking (%) *	607 (82.3 )	79.5-85.0	369 (75.3)	71.5-79.1	976 (79.5)
Risky drinking † (%) *	485 (79.9)	76.7-83.1	230 (62.3)	57.4-67.3	715 (73.3)
Physically active † (%)*	163 (21.4)	18.5-24.4	168 (33.8)	29.6-38.0	331 (26.3)
GGT ≥50 U/L (%) *	312 (42.7)	39.1-46.3	128 (26.2)	22.3-30.1	440 (36.1)
Diabetes (%)*	72 (9.5)	7.3-11.6	83 (16.7)	13.4-20.0	155 (12.3)
Metabolic syndrome † (%) *	199 (26.2)	23.1-29.3	232 (46.7)	42.3-56.1	431 (34.3)
Adverse MR cluster profile † (%) *	275 (40.8)	37.1-44.5	241 (50.6)	46.1-55.1	516 (44.9)

*significant difference of <0.05 between Aboriginal and TSI, using Chi square tests for categorical variables, t-test (for continuous variables with normal distribution, and Mann-Whitney rank-sum test for continuous variables with non-normal distribution‡. † Abdominal obesity defined using WHO WC gender specific criteria: overweight being WC of 80-88 cm in females and 94-102 cm in males, while obesity being WC ≥88 cm in females and 102 cm in males. Risky drinking was defined as > 6 drinks on any occasion or >4 drinks per day in males and >4 drinks on any occasion or >2 drinks per day in female in the week prior to the survey. Physically active, defined by American Heart Association criteria in which "active" means doing moderate to vigorous physical activity for more than 30 min per day for 5 days in the week before the survey. Metabolic syndrome defined by IDF criteria: waist circumference (≥94 cm in males and ≥80 for females), raised triglycerides (≥1.7 mmol/L), reduced HDL (<1.03 in males or <1.29 in females), raised blood pressure (systolic >130 mmHg or diastolic≥85 mmHg), and plasma glucose (≥5.6 mmol/L). Adverse MR (metabolic risk) cluster profile was obtained by EM cluster analysis. WC is waist circumference, Trigs is serum triglycerides, SBP is systolic blood pressure, DBP is diastolic blood pressure, HDL is high density lipoprotein cholesterol, FPG is fasting plasma glucose.

Table 2 describes the demographic, behavioral and metabolic characteristics of the cohort by normal and elevated GGT. The overall prevalence of elevated GGT was 25.9%. In this group, people were older, more likely to be male, Aboriginal, obese, smokers, drinkers, diabetic, be classified as metabolic syndrome, have an adverse MR cluster profile, and be physically inactive, when compared to those with normal GGT.

**Table 2:** Demographic and health risk factor status, by GGT category in Indigenous adults

	GGT<50 U/L (n = 1876)		GGT≥50 U/L (n = 657)		P*
	**Mean or No**.	95% CI	No. (%)	95% CI	
**Age (Years) (SD)**	36.6 (16.3)	35.8-37.3	39.3 (12.5)	38.4- 40.2	<0.01
**Male (%)**	779 (41.5)	39.3-43.8	440 (67.0)	63.4-70.6	<0.01
**Aborigines (%)**	1093 (58.3)	56.0-60.5	493 (75.0)	71.7-78.4)	<0.01
**WC (SD)**	94.3 (17.2)	93.5- 95.1	97.4 (14.5)	96.3-98.5	<0.01
**Abdominal obesity (%) †**					0.09
Normal	655 (34.9)	32.8-37.1	208 (31.7)	28.1-35.2	
Overweight	300 (16.0)	14.3-17.7	127 (19.3)	16.3-22.4	
Obesity	921 (49.1 )	46.8-51.4	322 (49.1)	45.2-52.8	
**BMI (SD)**	27.5 (7.2)	27.2- 27.9	27.5 (6.5)	27.0- 28.0	0.45
**BMI**					0.01
<25	760 (40.5)	38.3-42.7	244 (37.1)	33.4-40.8	
25-30	480 (25.6)	23.6-27.6	207 (31.5)	28.0-35.1	
30+	636 (33.9)	31.8-36.0	206 (31.4)	27.8-34.9	
**SBP (SD)**	128.2 (19.7)	127.3-129.1	135.4 (18.4)	134.0-136.8	<0.01
**DBP (SD)**	69.4 (13.1)	68.8-70.0	77.3 (13.2)	76.3- 78.3	<0.01
**HDL (SD)**	1.14 (0.3)	1.13-1.15	1.19 (0.4)	1.16-1.22	<0.01
**Smoking (%)**	982 (52.7)	50.4-55.0	460 (70.5)	66.9-73.9	<0.01
**Drinking (%)**	1117 (61.7)	59.5-64.0	565 (87.7)	85.2-90.3	<0.01
Risky drinking † (%) *	679 (60.8)	57.9-6365	466 (82.5)	79.3-85.6	<0.01
**Physically active †**	467 (24.9)	22.9-26.9	130 (19.8)	16.7-26.8	0.01
**FPG (SD)**	5.5 (2.5)	5.4-5.6	6.2 (2.9)	5.9- 6.4	<0.01
**Diabetes (%)**	236 (12.6)	11.1-14.1	133 (20.2)	17.2-23.3	<0.01
Trigs (SD) ‡*	1.5 (1.1)	1.4-1.52	2.7 (2.0)	2.5- 2.8	<0.01
**Metabolic syndrome (%) †**	683 (36.4)	34.2-38.6	346 (52.6)	48.8-56.5	<0.01
**Adverse MR Cluster profile (%) †**	570 (32.8)	30.6-35.0	384 (60.0)	56.2-63.8	<0.01

*significant difference of <0.05 between normal (<50 U/L) and elevated (≥50 U/L) GGT, using Chi square tests for categorical variables, t-test (for continuous variables with normal distribution, and Mann-Whitney rank-sum test for continuous variables with non-normal distribution‡. † Abdominal obesity defined using WHO WC gender specific criteria: overweight being WC of 80-88 cm in females and 94-102 cm in males, while obesity being WC ≥88 cm in females and 102 cm in males. Risky drinking was defined as > 6 drinks on any occasion or >4 drinks per day in males and >4 drinks on any occasion or >2 drinks per day in female in the week prior to the survey. Physically active, defined by American Heart Association criteria in which "active" means doing moderate to vigorous physical activity for more than 30 min per day for 5 days in the week before the survey. Metabolic syndrome defined by IDF criteria: waist circumference (≥94 cm in males and ≥80 for females), raised triglycerides (≥1.7 mmol/L), reduced HDL (<1.03 in males or <1.29 in females), raised blood pressure (systolic >130 mmHg or diastolic≥85 mmHg), and plasma glucose (≥5.6 mmol/L). Adverse MR (metabolic risk) cluster profile was obtained by EM cluster analysis. WC is waist circumference, Trigs is serum triglycerides, SBP is systolic blood pressure, DBP is diastolic blood pressure, HDL is high density lipoprotein cholesterol, FPG is fasting plasma glucose.

**Figure 1:**
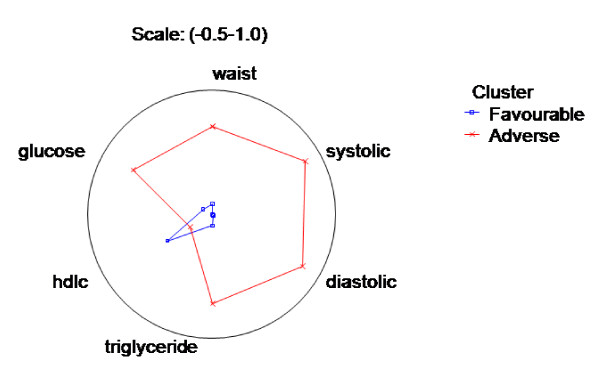
**A standardized comparison of metabolic risk factors between distinct Indigenous population clusters**. This radial/spider plot clearly shows the differences in metabolic risk factors between clusters. In the favorable compared to the adverse cluster, people have higher HDL levels and lower systolic and diastolic blood pressures, triglycerides, glucose and waist circumference. Data are shown in standardized units so that the magnitude of the difference between clusters can be compared between variables.

The MR cluster profile of the population is shown in table 3 and represented in standardized units in figure 1. There were two clusters identified in this population, denoted as 'favorable', which represented 60% of the population, and 'adverse' (40%). All metabolic variables were significantly worse and there were a significantly higher proportion of TSI people in the adverse cluster, when compared with the favorable cluster. The major contributors to cluster membership in descending order were: SBP, DBP, waist circumference, fasting glucose and triglycerides. HDL contributed little to explaining cluster membership. Together these variables explained 64% of individual allocation to each particular cluster.

**Table 3:** Metabolic risk cluster profiles of Indigenous adults across the studied rural far north Queensland communities

	Favorable cluster (n = 1430, 60%)		Adverse cluster (n = 955, 40%)	
	**Mean or No**.	SD	**Mean or No**.	SD
Male *	634 (44.3%)		516 (54.0%)	
Aboriginal *	912 (63.8%)		543 (56.9%)	
Age ( years)*	31.8	13.5	45.6	14.2
WC (cm)*	89.10	14.76	104.63	14.48
Trigs (mmol/L)‡*	1.25	0.65	2.65	1.96
SBP (mmHg)*	120.24	12.63	145.53	18.25
DBP (mmHg)*	64.80	9.61	81.56	12.26
HDL (mmol/L)*	1.19	0.32	1.09	0.27
FPG (mmol/L)*	4.73	0.76	7.23	2.61

*significant difference of <0.001 between favorable and adverse MR cluster, using t-test (for continuous variables with normal distribution, and Mann-Whitney rank-sum test for continuous variables with non-normal distribution‡. WC is waist circumference, Trigs is serum triglycerides, SBP is systolic blood pressure, DBP is diastolic blood pressure, HDL is high density lipoprotein cholesterol, FPG is fasting plasma glucose.

Disagreement between metabolic syndrome and MR cluster membership occurred in 524 cases; 286 cases in the favorable cluster were classified as having metabolic syndrome, whereas 238 in the adverse cluster were not. Aboriginal people when compared to TSI were more likely to be in the adverse cluster but not be classified as having metabolic syndrome (Additional file 2, Tables 1 and 2).

The multiple adjusted odds of elevated GGT were significantly greater in moderate and risky drinkers compared with non-drinkers (moderate: OR 2.3 [95%CI 1.6 - 3.2]; risky: OR 6.0 [4.4 - 8.2]), metabolic syndrome compared with no metabolic syndrome (OR 2.7 [2.1 - 3.5]), adverse compared with favorable MR cluster (OR 3.4 [2.6 - 4.3]), diabetics compared with non-diabetics (OR 2.1 [1.6 - 2.9]), and central and general overweight (central: OR 2.3 [1.6 - 3.2]; general: OR 1.7 [1.4 - 2.2]) and obesity (central: OR 3.7 [2.5 - 5.6]; general: OR 1.7 [1.3 - 2.2]), when compared to normal weight.

Ethnicity and sex stratified analyses demonstrated strong interactions between drinking and ethnicity and drinking and sex, on the odds of elevated GGT. Aboriginal drinkers were significantly more likely to have elevated GGT than TSI drinkers (OR 2.2 [1.8 - 2.8], Table 4), and male drinkers were twice as likely to have elevated GGT as female drinkers (OR 2.0 [1.2 - 3.5], Table 5). Moderate and risky alcohol drinking did not interact with ethnicity or sex on the odds of elevated GGT. There were no observed interactions between metabolic risk and either ethnicity or sex on elevated GGT.

**Table 4:** Alcohol drinking and metabolic risk versus elevated GGT (GGT ≥ 50 U/L) by ethnicity

	Aboriginal		TSI	
	Crude OR	Adj. OR*	Crude OR	Adj. OR*
**Alcohol Drinking**				
No	1.0	1.0	1.0	1.0
Yes	4.9 (3.6-6.7)	5.6 (3.9-8.0)	3.3 (2.1- 5.1)	2.2 (1.3-3.7)
**Alcohol risk**				
No	1.0	1.0	1.0	1.0
Moderate	2.1 (1.4-3.2)	2.7 (1.7-4.1)	1.9 (1.1-3.2)	1.4 (0.8-2.5)
Risky	6.3 (4.6-8.7)	7.6 (5.2-11.1)	4.5 (2.9-7.1)	3.4 (1.9-5.9)
**Metabolic syndrome †**				
No	1.0	1.0	1.0	1.0
Yes	2.1 (1.7-2.6)	2.7 (2.0-3.6)	2.8 (2.0-4.0)	2.2 (1.4-3.4)
**MR cluster profile †**				
Favorable	1.0	1.0	1.0	1.0
Adverse	3.3 (2.6-4.1)	3.4 (2.5-4.5)	3.9 (2.7-5.7)	3.2 (2.0-5.0)

* Adjusted for age, sex, ethnicity, BMI, smoking, and PA level. † Risky drinking was defined as > 6 drinks on any occasion or >4 drinks per day in males and >4 drinks on any occasion or >2 drinks per day in female in the week prior to the survey. Metabolic syndrome defined by IDF criteria: waist circumference (≥94 cm in males and ≥80 for females), raised triglycerides (≥1.7 mmol/L), reduced HDL (<1.03 in males or <1.29 in females), raised blood pressure (systolic >130 mmHg or diastolic≥85 mmHg), and plasma glucose (≥5.6 mmol/L). Adverse MR (metabolic risk) cluster profile was obtained by EM cluster analysis.

**Table 5:** Alcohol drinking and metabolic risk versus elevated GGT (GGT ≥ 50 U/L) by gender

	Female		Male	
	Crude OR	Adj. OR*	Crude OR	Adj. OR*
**Alcohol drinking**				
No	1.0	1.0	1.0	1.0
Yes	2.7 (1.9-3.8)	3.5 (2.3-5.2)	5.4 (3.6-8.0)	5.9 (4.0-9.9)
**Alcohol risk**				
No	1.0	1.0	1.0	1.0
Moderate	1.0 (0.6-1.9)	1.5 (0.9-2.6)	2.8 (1.8-4.5)	3.5 (2.1-5.9)
Risky	4.1 (2.9-5.8)	5.2 (3.4-8.0)	6.7 (4.4-10.0)	8.5 (5.3-13.5)
**Metabolic syndrome †**				
No	1.0	1.0	1.0	1.0
Yes	2.7 (2.0-3.7)	3.1 (2.2-4.5)	2.1 (1.6-2.6)	1.8 (1.3-2.5)
**MR cluster profile †**				
Favorable	1.0	1.0	1.0	1.0
Adverse	2.9 (2.1-3.9)	3.2 (2.2-4.7)	3.0 (2.3-3.8)	3.2 (2.3-4.4)

* Adjusted for age, ethnicity, BMI, smoking, and PA level. † Risky drinking was defined as > 6 drinks on any occasion or >4 drinks per day in males and >4 drinks on any occasion or >2 drinks per day in female in the week prior to the survey. Metabolic syndrome defined by IDF criteria: waist circumference (≥94 cm in males and ≥80 for females), raised triglycerides (≥1.7 mmol/L), reduced HDL (<1.03 in males or <1.29 in females), raised blood pressure (systolic >130 mmHg or diastolic≥85 mmHg), and plasma glucose (≥5.6 mmol/L). Adverse MR (metabolic risk) cluster profile was obtained by EM cluster analysis.

When stratified by drinking status (Table 6), there was no evidence of a multiplicative effect of moderate or risky drinking with obesity or metabolic syndrome on elevated GGT (interaction terms not shown). However, in people with an adverse MR cluster profile, drinking alcohol at risky levels was associated with a near 3-fold increase in the log of the odds of elevated GGT when compared with non-drinkers (OR 2.8 [1.6 - 4.9]).

**Table 6:** Risk of elevated GGT with obesity, metabolic syndrome and metabolic risk cluster by drinking status

	Non-drinkers (n = 791)		Moderate drinkers (n = 552)		Risky Drinkers † (n = 1183)	
	Crude OR	Adj. OR*	Crude OR	Adj. OR*	Crude OR	Adj. OR*
**Abdominal Obesity †**						
Normal	1.0	1.0	1.0	1.0	1.0	1.0
Overweight	1.4 (0.6-3.5)	1.5 (0.6-3.9)	1.5 (0.8-2.9)	1.9 (0.9-3.8)	1.8 (1.3-2.5)	3.2 (2.2-4.7)
Obese	2.0 (1.0-4.0)	2.6 (1.1-5.7)	1.7 (1.03-2.9)	4.5 (2.3-8.6)	1.5 (1.2-1.9)	4.2 (2.9-5.9)
**BMI**						
<25	1.0	1.0	1.0	1.0	1.0	1.0
25-30	2.0 (1.1-3.8)	2.0 (1.01-3.9)	2.1 (1.2-3.6)	3.0 (1.6-5.6)	1.5 (1.2-2.0)	2.4 (1.7-3.3)
30+	1.5 (0.8-2.8)	1.8 (0.9-3.6)	1.6 (0.9- 2.8)	2.7 (1.4-5.2)	1.4 (1.0-1.8)	2.9 (2.1-4.1)
**Metabolic syndrome †**						
No	1.0	1.0	1.0	1.0	1.0	1.0
Yes	2.3 (1.4-3.8)	2.4 (1.3-4.1)	2.5 (1.6-3.9)	2.8 (1.7-4.6)	2.8 (2.2-3.5)	3.7 (2.8-5.0)
**MR Cluster profile†**						
Favorable	1.0	1.0	1.0	1.0	1.0	1.0
Adverse	1.9 (1.2-3.1)	1.6 (0.9-2.8)	3.0 (1.9-4.8)	2.8 (1.6-4.9)	5.1 (3.9-6.6)	4.9 (3.7-6.7)

*Adjusted by age, sex, and ethnicity, smoking, and PA level. † Risky drinking was defined as > 6 drinks on any occasion or >4 drinks per day in males and >4 drinks on any occasion or >2 drinks per day in female in the week prior to the survey. Abdominal obesity defined using WHO WC gender specific criteria: overweight being WC of 80-88 cm in females and 94-102 cm in males, while obesity being WC ≥88 cm in females and 102 cm in males. Metabolic syndrome defined by IDF criteria: waist circumference (≥94 cm in males and ≥80 for females), raised triglycerides (≥1.7 mmol/L), reduced HDL (<1.03 in males or <1.29 in females), raised blood pressure (systolic >130 mmHg or diastolic≥85 mmHg), and plasma glucose (≥5.6 mmol/L). Adverse MR (metabolic risk) cluster profile was obtained by EM cluster analysis.

A *post-hoc *analysis was performed in order to investigate any possible interfering effects of HDL on the outcomes of the planned analysis, given that increases in both HDL (improvement) and GGT (worsening) result from increased alcohol consumption. Thus, we re-performed all steps of the above analysis, beginning with cluster analysis, excluding HDL. This process yielded two clusters of similar proportions to our previous analysis, the variable means and the proportions of Aboriginal and Torres Strait Islanders between clusters were similar to the original analysis (data not shown). Re-analysis of the individual effect of cluster membership on elevated GGT gave similar results (OR 3.5 [95% CI 2. 9 - 4.2] compared with OR 3.4 [2.6 - 4.3] in the original analysis) and re-analysis of table 6 showed very similar results to those of the original analysis, even after adjustment for HDL. The overall effect of the cluster × alcohol interaction term was also similar to the original analysis (OR 1.7 [95% CI 1.0 - 3.0]). When adjusted for HDL, the effect of the interaction term was essentially unchanged (OR 1.8 [95% CI 1.1 - 3.2]).

## Discussion

This study reported high population mean serum GGT in both men (61.0 ± 74.2 U/L) and women (35.0 ± 40.4 U/L) that exceeded the highest population mean concentrations published previously by a study in north-west Russia (Men: 43.8 ±60.5 U/L; Women: 28.3 ± 38.9 U/L) [29].

Having an adverse metabolic profile was associated with a 2.6 to 4.3-fold increase, and metabolic syndrome with a 2.1 to 3.5-fold increase, in the odds of having elevated GGT (≥50 U/L). A similar result for metabolic syndrome has been shown within abstainers in this cohort [13]. In this Indigenous population, an adverse metabolic profile conferred 3 times the risk of elevated GGT in risky drinkers than in abstainers. Interestingly, this finding did not hold when using the metabolic syndrome classification in place of the MR cluster. Previous studies in moderate alcohol drinkers and abstainers have claimed additive effects of alcohol consumption and BMI on elevated serum GGT and ALT [14,15], but an interactive effect modeled using logistic regression was not evident in this Australian indigenous population.

These findings are novel and are significant in this population. Although there are well known interactions between BMI and drinking on GGT, to the authors knowledge no published data exist regarding the interaction between multiple co-existing metabolic risk factors or different metabolic endophenotypes and alcohol intake on liver enzymes. This is important as the interactions may be much more severe for people with cardio-metabolic risk factors over and above overweight/obesity. Moreover, we have tested the interactions with two different methods for classifying cardio-metabolic risk: (1) the IDF metabolic syndrome definition; (2) a data driven approach to defining population metabolic endophenotypes using the same variables. This departure from the controversial dichotomous classification of the metabolic syndrome in capturing the population distribution of multiple metabolic risk factors is a major strength of this study. A priori, we conceived that internationally used clinical cut-points for metabolic syndrome classification may not apply equally to Australian Aboriginal and Torres Strait Islander peoples as there metabolic endophenotypes are quite different (as evidenced in this cohort by Table 1) and thus the relations between cardio-metabolic risk factors may vary. We indeed demonstrated that there was a discrepancy (n = 524 cases) between clinical metabolic syndrome classification and metabolic endophenotypes (clusters) that differed by ethnicity. Aboriginals were more likely to be classified as not having metabolic syndrome but being in the adverse MR cluster compared with Torres Strait Islanders who were more likely to be classified as metabolic syndrome but be in the favourable MR cluster. This could be partially due to inappropriate waist circumference cut-offs causing misclassification of IDF metabolic syndrome which may in turn partly explain the lack of interaction of alcohol drinking with metabolic syndrome and obesity on elevated GGT. MR clusters differed in their ethnic distribution, and cluster membership was mostly explained by blood pressure rather than abdominal obesity, which tends to explain clustering in Caucasian populations. These different metabolic (endo)phenotypes and their risk to future health outcomes are deserving of further study. The interactions of metabolic profile or body fat with alcohol consumption on serum GGT may be dependent on gene polymorphisms in the population that affect the level of fat accumulation in liver cells, oxidative stress, cytokine production, immune response and tissue fibrosis [30]. However, at present no genetic associations with advanced NAFLD have been confirmed [31].

This is the first study of the associations of alcohol intake and metabolic risk, their combined influence and the potentially modifying role of gender and ethnic background on elevated serum GGT in Indigenous Australians. Although we have only studied an intermediate outcome of liver function (GGT), to date very little has been published on biochemical liver dysfunction or NAFLD in Indigenous populations worldwide. The prevalence of elevated GGT in the communities studied (overall 25.9%: males 36.1%, females 16.5%) were comparable to those reported in the Russian study. Similarly, in an Atayal Aboriginal community in Taiwan, the overall prevalence of elevated GGT (defined in this study as ≥61 U/L) was 22.4% (males 34.9%, females 10.7%). Over two thirds of this population were Aboriginal and had an elevated GGT prevalence of 28.6% compared with 8.8% in non-aboriginals [32]. It has been suggested that the high prevalence of type 2 diabetes in North American indigenous populations may indicate a high prevalence of NAFLD [33], and despite a lack of published data, this may also be true in Indigenous Australians. Elevated serum GGT and diagnosed NAFLD have also been shown to predict incident cardiovascular events in the general population [34], and more strongly in diabetics [34,35] and alcohol drinkers [34].

Almost 80% of Indigenous males and 59% of Indigenous females in this study reported that they currently drink alcohol. These percentages appear higher than Australian population estimates for Indigenous Australians generally [36] and reflects near universal access to alcohol from "wet" canteens in these remote communities in far north Queensland at the time of the study. In these communities, drinking alcohol was associated with a 3.6 to 6.2-fold increase in the chance of having elevated GGT after adjustment for age, gender and ethnicity and the effect increased from moderate to risky alcohol consumption. The association between alcohol intake and GGT is well described [11] with modifications of this association by sex [37], ethnicity [38-40] and age [41]. In these communities, the observed doubling in risk of elevated GGT from drinking in males compared with females, and in Aboriginal compared with TSI peoples was directly related to the prevalence of high risk alcohol consumption by males compared with females and Aboriginal compared with TSI people. Thus, irrespective of sex or ethnicity, people who drink alcohol at risky levels have equivalent odds of elevated GGT.

Limitations to this study include a possible downward response bias to 7-day recall of alcohol consumption [42], the sole use of GGT as an outcome and indicator of NAFLD due to the limited ability to perform more advanced investigations in very remote communities, and a small degree of missing data on major exposure variables that differentially occurred in participants who were female, older and had lower blood pressure.

## Conclusions

Taken together, drinking was most strongly associated with elevated GGT in a dose dependent manner. Aboriginal men were at particularly high risk of elevated GGT from drinking, due largely to the high prevalence of risky drinking in this group. In this Indigenous population, an adverse metabolic profile conferred 3 times the risk of elevated GGT in risky drinkers than in abstainers, independent of sex and ethnicity. Interventions need to target modifiable community determinants of both the population's metabolic risk status and risky alcohol drinking behaviour to achieve greater reduction in population GGT levels. The effects of such strategies on the rates of new diabetes, CHD and stroke cases (major contributors to the Indigenous health gap in Australia) require further investigation.

## Competing interests

The authors declare that they have no competing interests.

## Authors' contributions

MTH contributed to the analysis and interpretation of data, drafting of the manuscript and critical revision of the manuscript for important intellectual content and data analysis. ML performed the data analysis and contributed to interpretation of data, drafting of the manuscript, critical revision of the manuscript for important intellectual content. JP performed data analysis and contributed to interpretation of data, drafting and critical revision of the manuscript for important intellectual content. RAM was involved in the conception and design of the study, acquisition of data, interpretation of data and critical revision of the manuscript for important intellectual content and for obtaining funding and administrative, technical and material support. ML had full access to all of the data in the study and takes responsibility for the integrity of the data and the accuracy of the data analysis. All authors read and approved the final manuscript.

## Pre-publication history

The pre-publication history for this paper can be accessed here:

http://www.biomedcentral.com/1471-2458/10/454/prepub

## Supplementary Material

Additional file 1 **Expectation Maximisation Cluster analysis and Partial Least Squares expanded methods**. This file contains an expanded discussion of the EM cluster and PLS analysis methods. Click here for file

Additional file 2 **Disagreement between IDF metabolic syndrome classification and MR cluster membership**. This file contains two tables that show (1) the level of disagreement between the metabolic syndrome classification and MR cluster membership, and (2) the demographic and metabolic characteristics of cases in which disagreement occurred. Click here for file
