# Supplementary material for: Alcohol, metabolic risk and elevated serum gamma-glutamyl transferase (GGT) in Indigenous Australians
Source: BMC Public Health. 2010 Aug 3;10:454. doi: 10.1186/1471-2458-10-454 (PMC2920283; doi:10.1186/1471-2458-10-454)
Supplement: Additional file 1 — Expectation Maximisation Cluster analysis and Partial Least Squares expanded methods. This file contains an expanded discussion of the EM cluster and PLS analysis methods. [file 1471-2458-10-454-S1.DOC]

**Additional File 1 - Expectation Maximisation Cluster analysis and Partial Least Squares expanded methods**

**METHODS**

**Derivation of the metabolic risk factor cluster variable (MR cluster)**

Cluster Analysis is the process of assigning members of a population into groups such that the members of each group share common characteristics. The method is an example of unsupervised learning. There is no *a priori* assumption made about what the groups represent or indeed the number of groups. The classical k-means cluster algorithm requires that the number of clusters to be extracted be specified beforehand. Clusters are then extracted so that the means across all clusters for all variables are as different as possible. The clusters can then be examined for differences. If these differences are not clear then the process can be repeated with a different number of clusters. This process is extended using the Expectation Maximization (EM) algorithm. Rather than looking at means, the membership for each case is established by a maximum likelihood technique where the probability of cluster membership is based on one or more probability distributions. The EM algorithm does not require the number of clusters to be pre-specified, but instead employs a cross validation technique (v-fold cross validation) to estimate the optimal number of clusters. The method involves drawing v random samples to which the model is applied. The training sample consists of v-1 samples and the results are applied to the remaining sample (the test sample). Over-fitting is avoided and superfluous clusters that do not add significant information are not fitted. EM clustering was applied to waist circumference, TG, HDL, systolic and diastolic blood pressure and fasting glucose and was performed in STATISTICA (data analysis software system), version 8.0 (StatSoft, Inc. (2007)).

To establish which variables best contribute to cluster membership Partial Least Squares (PLS) was applied. The outcome variable was cluster membership number, a multinomial variable. PLS was used since the predictors (the original variables) are highly correlated and so ordinary multinomial regression is inaccurate due to multicollinearity. The set of n correlated independent variables undergoes dimension reduction and a set of p (< n) components are extracted. These components consist of a linear combination of the independent variables with different loadings within each component. The Non-Iterative Partial Least Squares (NIPALS) version of PLS was used (<http://www.vias.org/tmdatanaleng/dd_nipals_algo.html>**)**. Cross validation was used to determine the optimal number of components which avoids over-fitting by removing non-significant components. Finally, the relative importance of each original variable can be established.
