# Supplementary material for: Alcohol, metabolic risk and elevated serum gamma-glutamyl transferase (GGT) in Indigenous Australians
Source: BMC Public Health. 2010 Aug 3;10:454. doi: 10.1186/1471-2458-10-454 (PMC2920283; doi:10.1186/1471-2458-10-454)
Supplement: Additional file 2 — Disagreement between IDF metabolic syndrome classification and MR cluster membership. This file contains two tables that show (1) the level of disagreement between the metabolic syndrome classification and MR cluster membership, and (2) the demographic and metabolic characteristics of cases in which disagreement occurred. [file 1471-2458-10-454-S2.DOC]

**Additional File 2 – Disagreement between IDF metabolic syndrome classification and MR cluster membership**

|  | **IDF metabolic syndrome** | |  |
| --- | --- | --- | --- |
| **Cluster** | No | Yes | Total |
| Favorable | 1144 | 286 | 1430 |
|  | *82.78* | *28.51* | *59.96* |
| Adverse | 238 | 717 | 955 |
|  | *17.22* | *71.49* | *40.04* |
| Total | 1382 | 1003 | 2385 |
|  | *100.00* | *100.00* | *100.00* |

**Table 1 Disagreement between the IDF definition of metabolic syndrome and MR cluster membership in an Indigenous population sample from rural far north Queensland communities.** Data presented are counts and *column percentages. 28.5%* of people in the ‘Favorable’ cluster were classified as metabolic syndrome and 17.2% of people in the ‘Adverse’ cluster did not classify as metabolic syndrome.

**Table 2 Demographic and metabolic characteristics of cases where disagreement between IDF metabolic syndrome and MR cluster occurred.**

|  | **Favourable MR cluster but IDF MetS case (n=286)** | | **Adverse MR cluster but non IDF MetS case (n=238)** | | **Total case disagreement (N=524)** | |  | |
| --- | --- | --- | --- | --- | --- | --- | --- | --- |
|  | **Mean or No.** | **SD** | **Mean or No.** | **SD** | **Mean or No.** | **SD** |  |  |
| **Male** | 81 (28.3%) |  | 181 (76.1%) |  | 262 (50.0%) |  |  | |
| **Aboriginal** | 158 (55.2%) |  | 172 (72.3) |  | 330 (63.0%) |  |  | |
| **TSI** | 128 (44.8) |  | 66 (27.7) |  | 194 (37.0%) |  |  | |
| **Age ( years)** | 36.5 | 14.8 | 43.9 | 14.1 | 39.9 | 14.9 |  | |
| **WC (cm)** | 100.5 | 11.3 | 93.8 | 14.1 | 97.4 | 13.1 |  | |
| **Trigs (mmol/L)** | 1.8 | 0.6 | 2.3 | 1.8 | 2.0 | 1.3 |  | |
| **SBP (mmHg)** | 124.5 | 12.5 | 145.2 | 19.4 | 133.9 | 19.0 |  | |
| **DBP (mmHg)** | 66.7 | 8.8 | 85.5 | 13.0 | 75.2 | 14.3 |  | |
| **HDL (mmol/L)** | 1.0 | 0.2 | 1.2 | 0.3 | 1.1 | 0.3 |  | |
| **FPG (mmol/L)** | 5.1 | 1.0 | 5.9 | 3.0 | 5.5 | 2.2 |  | |

WC is waist circumference, Trigs is serum triglycerides, SBP is systolic blood pressure, DBP is diastolic blood pressure, HDL is high density lipoprotein cholesterol, FPG is fasting plasma glucose.
